# Editorial Comment: Effects of testicular dysgenesis syndrome components on testicular germ cell tumor prognosis and oncological outcomes

**DOI:** 10.1590/S1677-5538.IBJU.2019.0387.1

**Published:** 2020-07-31

**Authors:** Andréia Cristina de Melo

**Affiliations:** 1 Instituto Nacional do Câncer do Brasil - INCA Divisão de Pesquisa Clínica Rio de Janeiro RJ Brasil Divisão de Pesquisa Clínica, Instituto Nacional do Câncer do Brasil - INCA, Rio de Janeiro, RJ, Brasil

## COMMENT

Testicular cancer is the most curable solid tumor and the most common malignancy in men between the ages of 18 and 35 years, although it accounts for just 1% of all cancers in men (1). Testicular germ cell tumors account for 95% of testicular cancers which are classified as either seminomas or non-seminomas (2).

There are a variety of known risk factors for testicular neoplasia, including cryptorchidism (3), history of hypospadias (4), individuals with androgen insensitivity syndrome or mixed gonadal dysgenesis (5, 6), a personal or family history of testicular cancer, infertility or subfertility (7) and HIV infection (8). All of these risk factors predispose to the development of carcinoma in situ and invasive testicular cancer.

Testicular dysgenesis syndrome composed by undescended testis, hypospadias, decreased spermatogenesis and testicular germ cell tumor has also been recently described (9). Thus far, there has been a lack of information regarding the effects of the testicular dysgenesis syndrome on the testicular germ cell tumor prognosis.

The current issue of the International Brazilian Journal of Urology presents an interesting original paper from a Turkish group. Selvi and colleagues on the paper entitled “Effects of testicular dysgenesis syndrome components on testicular germ cell tumor prognosis and oncological outcomes” (10) retrospectively assessed the clinical characteristics and oncological outcomes of 69 patients who underwent radical orchiectomy due to testicular germ cell tumor. In a subgroup analysis, higher testicular dysgenesis syndrome rates were found in advanced stage testicular tumors (36.1% versus 9.1%; p=0.008). The group with testicular dysgenesis syndrome had higher local recurrence, distant metastasis, and also higher cancer-specific mortality in comparison to the group without the syndrome (the differences were statistically significant, p<0.001 for the 3 outcomes). In terms of survival, the recurrence-free survival, the metastasis-free survival and the cancer-specific survival were statistically significant lower in the group of patients with testicular dysgenesis syndrome. In the multivariate analysis, testicular dysgenesis syndrome was the most important independent predictive factor related with local recurrence, distant metastasis, recurrence-free survival, metastasis-free survival and cancer-specific survival in both seminomas and non-seminomas, and also for the entire group of patients diagnosed with testicular germ cell tumor.

Despite the originality of this scientific report, some limitations must be addressed, since it is based on a small retrospective single center cohort, susceptible to selection bias once the patients whose data could not be completed were excluded from the study.

Finally, this is a thought-provoking hypothesis which is generating research. Further studies are warranted in order to confirm the current findings.
